# Sex differences in risk factors for coronary heart disease events: a prospective cohort study in Iran

**DOI:** 10.1038/s41598-023-50028-0

**Published:** 2023-12-16

**Authors:** Azra Ramezankhani, Fereidoun Azizi, Farzad Hadaegh

**Affiliations:** 1 Prevention of Metabolic Disorders Research Center, Research Institute for Endocrine Sciences, Shahid Beheshti University of Medical Sciences, Tehran, Iran; 2 Endocrine Research Center, Research Institute for Endocrine Sciences, Shahid Beheshti University of Medical Sciences, Tehran, Iran

**Keywords:** Cardiology, Diseases, Medical research, Risk factors

## Abstract

We investigated sex-specific associations and their differences between major cardiovascular risk factors and the risk of incident coronary heart disease (CHD) and hard CHD (defined as nonfatal myocardial infarction and CHD death). A total of 7518 (3377 men) participants from the Tehran Lipid and Glucose Study were included. Cox models were used to estimate the hazard ratios (HRs) and women-to-men ratios of HRs for CHD events associated with each risk factor. During 20 years of follow-up (1999–2018), 1068 (631 men) and 345 (238 men) new cases of CHD and hard CHD, respectively, were documented. In total population, the incidence rates per 1000 person-years were 9.5 (9.0–10.1) and 2.9 (2.6–3.2) for CHD and hard CHD, respectively. Hypertension, diabetes, pre-diabetes, and a high waist-to-hip ratio (WHR) were associated with a greater HR of hard CHD in women than men; the women-to-men HRs were 2.85 [1.36–5.98], 1.92 [1.11–3.31], 2.04 [1.09–3.80] and 1.42 [1.10–1.82], respectively. Diabetes was associated with a higher HR of CHD in women than men (ratio of HRs 1.49 (1.10–2.01). In conclusion, we found that hypertension, diabetes, pre-diabetes, and high WHR conferred a greater excess risk of CHD events in women than in men, suggesting that Iranian women may require greater attention for the prevention of CHD events.

## Introduction

Coronary heart disease (CHD) is recognized as the primary contributor to morbidity and mortality worldwide, responsible for around 17% of global deaths in 2016^1^. At younger ages, men have a greater risk of CHD than women, but this gender disparity diminishes as individuals grow older. For example, middle-aged men exhibit a 4 to fivefold greater risk of developing CHD in comparison to women, but this ratio decreases by 2 times after that^2^.

Clinical studies have shown that women typically experience the onset of heart disease approximately a decade later than men^3^. Additionally, sex differences have been observed in the clinical presentation, symptoms, treatments, and outcomes of CHD. Some of these differences are due to the effect of sex-specific risk factors; and also the effect and prevalence of common risk factors^4,5^. Several large-scale meta-analytic investigations have revealed clinically meaningful differences between sexes in the impact of certain traditional risk factors on the risk of developing CHD. For instance, these meta-analyses have indicated that diabetes^6^ and current smoking^7^ were more strongly associated with CHD in women compared to men. However, the published meta-analyses have a substantial heterogeneity in the population, study design, statistical analysis, and adjustment for confounders. Furthermore, current meta-analysis studies have primarily focused on examining a single risk factor and did not consider sex differences for a wide range of risk factors^6–8^.

Despite the growing prevalence of CHD in the Middle East and North Africa (MENA) region^9,10^, there is a lack of research on the sex differences in major CHD risk factors. Previous cohort studies conducted in the MENA region have primarily focused on examining the association between major risk factors and CHD separately for men and women, without comparing the relative risk (RR) between the sexes for each risk factor^11–14^. Therefore, the objective of this study was to evaluate the sex-specific association between major cardiovascular disease (CVD) risk factors and the occurrence of CHD and hard CHD events among Iranian adults participating in the population‐based cohort of the Tehran Lipid and Glucose Study (TLGS). Additionally, the study aimed to compare the RRs between men and women for these risk factors.

## Methods

### Study population

The TLGS is a large, prospective, population-based cohort study that was initiated in 1999 to identify the risk factors and outcomes associated with non-communicable diseases (NCDs). Detailed information regarding the sampling methods and design of the TLGS can be found in previously published literature^15^. In summary, participants of TLGS were recruited in two distinct phases: the first phase took place between 1999 and 2001, while the second phase occurred between 2002 and 2005^15^. For the current study, 9553 participants aged ≥ 30 years were selected from the first (n = 7927) and second (n = 1626) phases. We excluded 602 individuals with a history of CVD, 783 people who did not have complete follow-up data until the end of the study, and 650 individuals who had missing value on study covariates including systolic blood pressure (SBP), diastolic blood pressure (DBP), body mass index (BMI), waist circumference (WC), waist-to-hip ratio (WHR), waist-to-height ratio (WHtR), diabetes status, smoking status, education status, high-density lipoprotein cholesterol (HDL-C), total cholesterol (TC), and fasting plasma glucose (FPG). These exclusions resulted in a base sample of 7518 (3377 men) participants who had completed follow-up data up to 20 March 2018, accounting for 84% of the eligible participants (Supplementary Fig. 1). The proposal of this study was approved by the ethics committee of the Research Institute for Endocrine Sciences of Shahid Beheshti University of Medical Sciences, Tehran, Iran, and all participants provided written informed consent. All methods employed in this study adhered to the relevant guidelines and regulations.

### Measurements

In the TLGS study, standardized questionnaires were used to collect information on participants' age, sex, education level, smoking status, medication use, history of CVD, and family history of premature CVD (FH-CVD). Anthropometric measurements, such as weight, height, WC, and hip circumference were taken following standard protocols. BMI was calculated as weight (kg)/height (m^2^); WHR was determined by dividing WC by hip circumference; while WHtR was calculated by dividing the WC by height. SBP and DBP were measured twice using a mercury column sphygmomanometer, and the average of two measurements was recorded as the participant's blood pressure (BP). Fasting Blood samples were collected from all participants to measure FPG, 2-h post-challenge plasma glucose (2 h-PCPG), TC, and HDL-C using the enzymatic colorimetric method^15^.

### Definition of terms

A positive FH-CVD for the participants was defined as any previously diagnosed CVD in a female first-degree relative younger than 65 years or in a male first-degree relative younger than 55 years. BP was categorized as follows: normal if SBP < 120 mm Hg and DBP < 80 mm Hg; pre-hypertension if SBP ranged from 120 to140 mm Hg or DBP ranged from 80 to 90 mm Hg; and hypertension if SBP was 140 mm Hg or higher, or DBP was 90 mm Hg or higher, or if the participant was taking antihypertensive drugs^16^. Normal weight, overweight, and obesity were defined as a BMI < 25 kg/m^2^, 25 ≤ BMI < 30 kg/m^2^, and ≥ 30 kg/m^2^, respectively. Central obesity was defined as a WC ≥ 95 cm for both sexes^17^. Diabetes status was categorized to no-diabetes: FPG < 5.55 mmol/L and 2-hPCPG < 7.77 mmol/L; pre-diabetes: 5.55 ≤ FPG < 7 mmol/L or 7.77 ≤ 2 h-PCPG < 11.1 mmol/L; diabetes: FPG ≥ 7 mmol/L or 2-hPCPG ≥ 11.1 mmol/L or the use of anti-diabetic drugs. Smoking status was categorized into: current smokers (daily or occasionally); no smokers: those who used to smoke and never smokers. Education was categorized into three levels: < 6 years, 6–12 years, and ≥ 12 years of education. Elevated TC was defined as a TC level of ≥ 6.216 mmol/L. Low HDL-C was defined as HDL-C < 1.036 mmol/L for men and < 1.295 mmol/L for women.

### Outcome events

The methodology for collecting cardiovascular outcomes in TLGS has been previously published^18^. Participants were followed from their inclusion in the TLGS study until March 20, 2018, or until the occurrence of their first CHD event. CHD events were defined as cases of definite myocardial infarction (MI) diagnosed through electrocardiogram (ECG) and biomarkers, probable MI diagnosed by positive ECG findings along with cardiac symptoms or signs but with biomarkers showing negative or inconclusive results, unstable angina pectoris, angiographic-proven CHD, and CHD-related death. Nonfatal MI and CHD-related death (including fatal MI) were categorized as hard CHD.

### Statistical methods

The baseline characteristics of the study population are presented as number (percentage) and mean [standard deviation (SD)], stratified by sex. Additionally, we conducted a comparison of the baseline characteristics between respondents (individuals with complete data at baseline and at least one follow-up) and non-respondents (individuals with missing data at baseline or no follow-up data).

The crude incidence rates and 95% confidence interval (CI) for each outcome were calculated per 1000 persons-years, separately for women and men.

The Cox regression models were used to estimate the hazard ratios (HRs) and 95% CIs for study outcomes for each risk factor in men and women. All models were adjusted for age, FH-CVD, and the use of antihypertensive and lipid-lowering drugs as well as a set of additional confounders specific to each risk factor. For BP-related variables, additional adjustments were made for smoking status, BMI, diabetes, education, and TC. Smoking status was further adjusted for SBP, BMI, diabetes, education, and TC. Diabetes-related variables were further adjusted for smoking status, BMI, SBP, education, and TC. Measures of body anthropometry were adjusted for smoking status, SBP, diabetes, education, and TC. Education was further adjusted for smoking status, BMI, diabetes, SBP, and TC. Lipid-related variables were adjusted for smoking status, BMI, diabetes, SBP, and education. In the above models, we included an interaction term between each risk factor and sex to estimate the women-to-men relative HR (ratio of HRs). For WHR and WHtR, the results are shown as the HR per sexes-combined 1-SD increase. For BMI, WC, and HDL-C, the results are shown for each 5-unit increase. We have also shown the HRs per each 20-unit increase for SBP and TC; and per each 10-unit increase for DBP and FBS. Analyses were performed using R version 4.1.2 (https://www.r-project.org), and a two-sided *P* value < 0.05 was considered statistically significant.

## Results

The baseline characteristics of the study population (n = 7518; 55% women) are presented in Table 1. The mean (SD) age at baseline was 47.5 (12.8) and 46.0 (11.3) in men and women, respectively. At baseline, a smaller proportion of men than women had hypertension, obesity, diabetes, and FH-CVD. In general, men had lower levels of DBP, BMI, WHtR, FPG, HDL-C and TC than women. Men were also more likely to be smokers and had higher levels of education compared to women.

**Table 1:** Baseline characteristics of the study population, Tehran Lipid and Glucose Study (1999–2018).

Characteristics	Men (n = 3377)	Women (n = 4141)
Age, year	47.50 (12.85)	46.02 (11.38)
Blood pressure, mm Hg		
SBP	121.31 (18.90)	121.09 (20.23)
DBP	78.35 (11.23)	78.79 (10.78)
Hypertension, n (%)		
No hypertension	1424 (42.2)	1680 (40.6)
Pre-hypertension	1194 (35.4)	1355 (32.7)
Hypertension	759 (22.5)	1106 (26.7)
BMI categories, n (%)		
Normal weight	1296 (38.4)	956 (23.1)
Overweight	1538 (45.5)	1711 (41.3)
Obese	543 (16.1)	1474 (35.6)
Body anthropometry		
BMI, kg/m^2^	26.23(3.94)	28.57 (4.76)
WC, cm	90.90 (10.82)	90.76 (12.07)
Waist-to-hip ratio	0.93 (0.06)	0.86 (0.08)
Waist-to-height ratio	0.53 (0.06)	0.58 (0.08)
WC categories, n (%)		
Normal WC	2121 (62.8)	2585 (62.4)
Elevated WC	1256 (37.2)	1556 (37.6)
Diabetes status, n (%)		
No diabetes	2201 (65.2)	2585 (62.4)
Pre-diabetes	769 (22.8)	964 (23.3)
Diabetes	407 (12.1)	592 (14.3)
FPG, mmol/l	5.53 (1.73)	5.62 (2.07)
Smoking status, n (%)		
Past and never	2311 (68.4)	3941 (95.2)
Current	1066 (31.6)	200 (4.8)
Education level, n (%)		
< 6 years	994 (29.4)	1923 (46.4)
6–12 years	1776 (52.6)	1898 (45.8)
> 12 years	607 (18.0)	320 (7.7)
Lipids categories, n (%)		
Normal HDL-C	1163 (34.4)	1056 (25.5)
Low HDL-C	2214 (65.6)	3085 (74.5)
Normal TC	2708 (80.2)	2908 (70.2)
Elevated TC	669 (19.8)	1233 (29.8)
Lipids measures, mmol/l		
HDL-C	0.97 (0.24)	1.15 (0.28)
TC	5.35 (1.09)	5.66 (1.22)
Drug use, n (%)		
Antihypertensive drugs	144 (4.3)	439 (10.6)
Lipid-lowering drugs	76 (2.3)	199 (4.8)
Family history of CVD, n (%)	477 (14.1)	750 (18.1)

Values are presented as mean (SD) or n (%).

*SBP* Systolic blood pressure, *DBP* Diastolic blood pressure, *BMI* Body mass index, *WHR* Waist-to-hip ratio, *WHtR* Waist-to-height ratio, *WC* Waist circumference, *FPG* Fasting plasma glucose, *HDL-C* High density lipoprotein cholesterol, *TC* Total cholesterol, *CVD* cardiovascular disease.

Supplementary Table 1 presents the comparisons between respondents and non-respondents. Generally, respondents had a higher level for all anthropometric indices and a lower level of HDL-C, compared with non-respondents. Additionally, respondents were less likely to be smokers than non-respondents.

The median follow-up was 17.9 (interquartile range: 13.8–18.4) years. During the study period, a total of 1068 (631 men) and 345 (238 men) new cases of CHD and hard CHD, respectively, were documented.

In the total study population, crude incidence rates (95% CI) per 1000 person-years were 9.5 (9.0–10.1) for CHD and 2.9 (2.6–3.2) for hard CHD. The corresponding values were 6.9 (6.3–7.6) in women and 13.1 (12.1–14.1) in men, for CHD events. For hard CHD, the values were 1.6 (1.3–1.9) in women and 4.6 (4.1–5.2) in men, respectively.

In confounders adjusted models, we found that the rate of CHD in women was less than half that in men (relative HR 0.46; 95% CI 0.40–0.53). The corresponding value was 0.34 (0.26–0.45) for hard CHD.

### Associations of risk factors with CHD events

**Figure 1:**
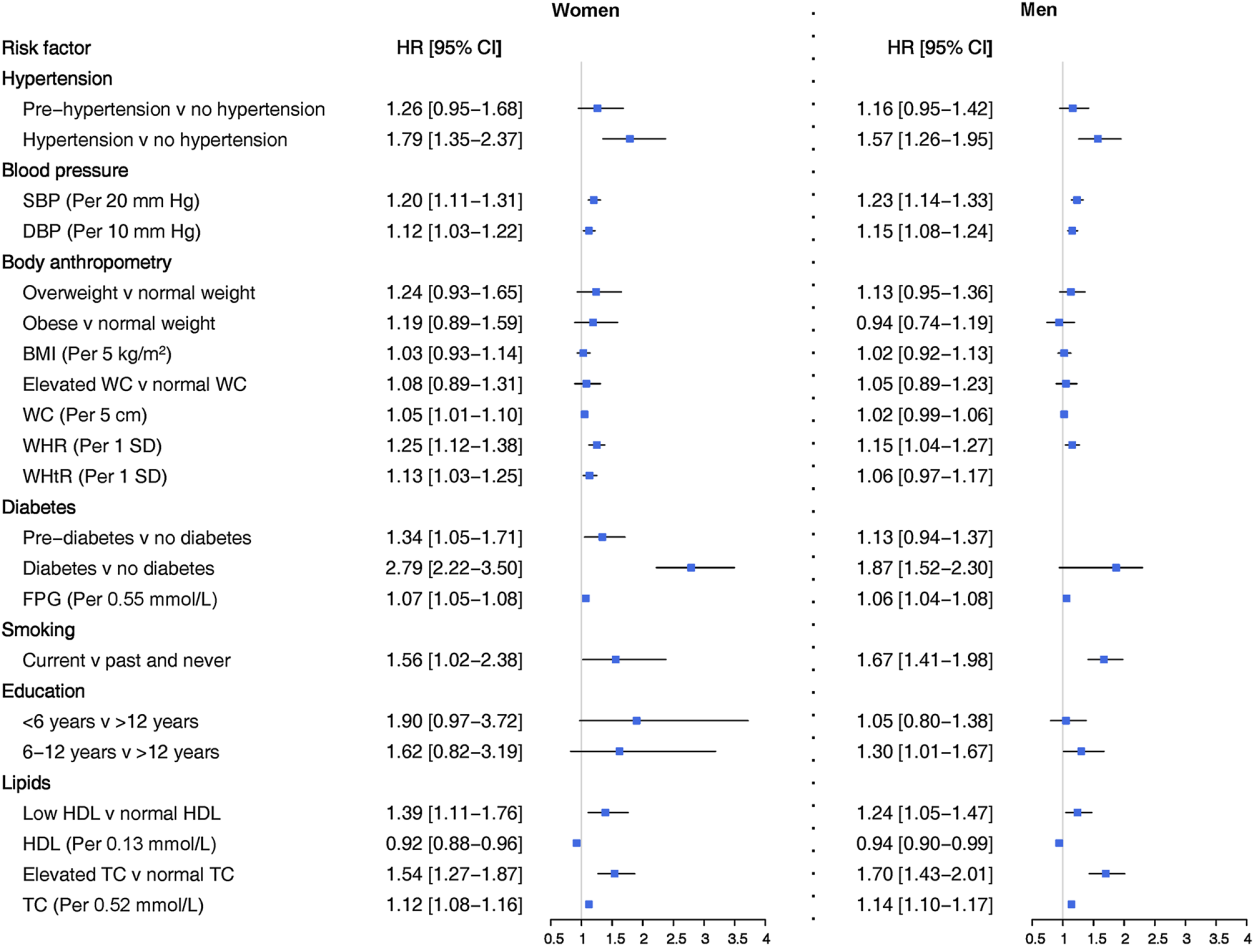
Adjusted hazard ratios for association between risk factors and incident CHD by sex. SBP: systolic blood pressure; DBP: diastolic blood pressure; BMI: body mass index; WHR: waist-to-hip ratio; WHtR: waist-to-height ratio; WC: waist circumference; FPG: fasting plasma glucose; HDL: high-density lipoprotein cholesterol; TC: total cholesterol. Blood pressure-related variables were adjusted for age, smoking status, BMI, diabetes, education, TC, antihypertensive and lipid-lowering drugs, and FH-CVD. Measures of body anthropometry were adjusted for age, smoking status, SBP, diabetes, education, TC, antihypertensive and lipid-lowering drugs, and FH-CVD. Diabetes-related variables were adjusted for age, smoking status, BMI, SBP, education, TC, antihypertensive and lipid-lowering drugs, and FH-CVD. Smoking was adjusted for age, BMI, diabetes, SBP, education, TC, antihypertensive and lipid-lowering drugs, and FH-CVD. Education was adjusted for age, smoking status, BMI, diabetes, SBP, TC, antihypertensive and lipid-lowering drugs, and FH-CVD. Lipid-related variables were adjusted for age, smoking status, BMI, diabetes, SBP, education, antihypertensive and lipid-lowering drugs, and FH-CVD.

**Figure 2:**
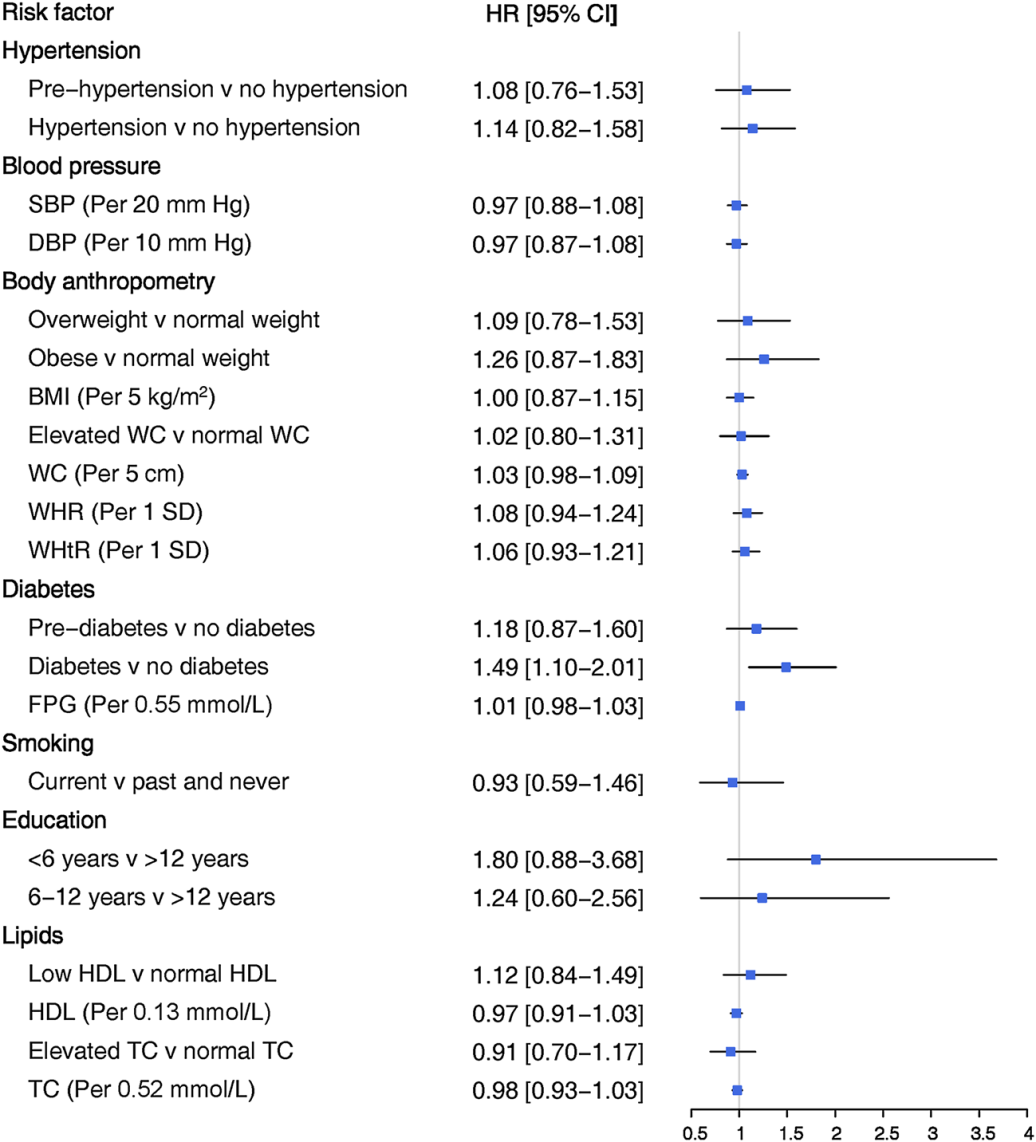
Adjusted women-to-men ratios of hazard ratios for association between risk factors and incident CHD. SBP: systolic blood pressure; DBP: diastolic blood pressure; BMI: body mass index; WHR: waist-to-hip ratio; WHtR: waist-to-height ratio; WC: waist circumference; FPG: fasting plasma glucose; HDL: high density lipoprotein cholesterol; TC: total cholesterol. Blood pressure-related variables were adjusted for age, smoking status, BMI, diabetes, education, TC, antihypertensive and lipid-lowering drugs, and FH-CVD. Measures of body anthropometry were adjusted for age, smoking status, SBP, diabetes, education, TC, antihypertensive and lipid-lowering drugs, and FH-CVD. Diabetes-related variables were adjusted for age, smoking status, BMI, SBP, education, TC, antihypertensive and lipid-lowering drugs, and FH-CVD. Smoking was adjusted for age, BMI, diabetes, SBP, education, TC, antihypertensive and lipid-lowering drugs, and FH-CVD. Education was adjusted for age, smoking status, BMI, diabetes, SBP, TC, antihypertensive and lipid-lowering drugs, and FH-CVD. Lipid-related variables were adjusted for age, smoking status, BMI, diabetes, SBP, education, antihypertensive and lipid-lowering drugs, and FH-CVD.

**Figure 3:**
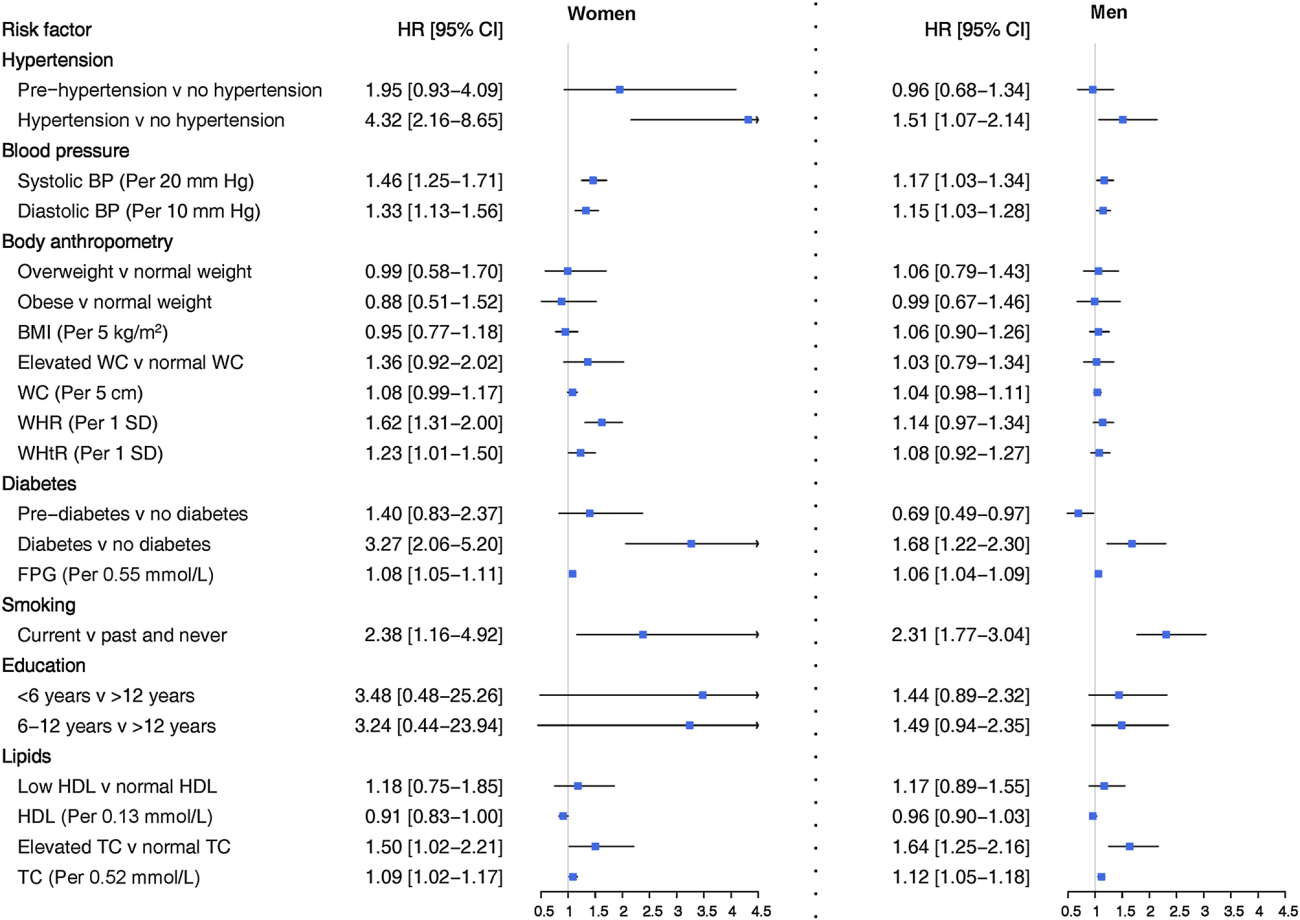
Adjusted hazard ratios for association between risk factors and incident hard CHD by sex. SBP: systolic blood pressure; DBP: diastolic blood pressure; BMI: body mass index; WHR: waist-to-hip ratio; WHtR: waist-to-height ratio; WC: waist circumference; FPG: fasting plasma glucose; HDL: high density lipoprotein cholesterol; TC: total cholesterol. Blood pressure-related variables were adjusted for age, smoking status, BMI, diabetes, education, TC, antihypertensive and lipid-lowering drugs, and FH-CVD. Measures of body anthropometry were adjusted for age, smoking status, SBP, diabetes, education, TC, antihypertensive and lipid-lowering drugs, and FH-CVD. Diabetes-related variables were adjusted for age, smoking status, BMI, SBP, education, TC, antihypertensive and lipid-lowering drugs, and FH-CVD. Smoking was adjusted for age, BMI, diabetes, SBP, education, TC, antihypertensive and lipid-lowering drugs, and FH-CVD. Education was adjusted for age, smoking status, BMI, diabetes, SBP, TC, antihypertensive and lipid-lowering drugs, and FH-CVD. Lipid-related variables were adjusted for age, smoking status, BMI, diabetes, SBP, education, antihypertensive and lipid-lowering drugs, and FH-CVD.

**Figure 4:**
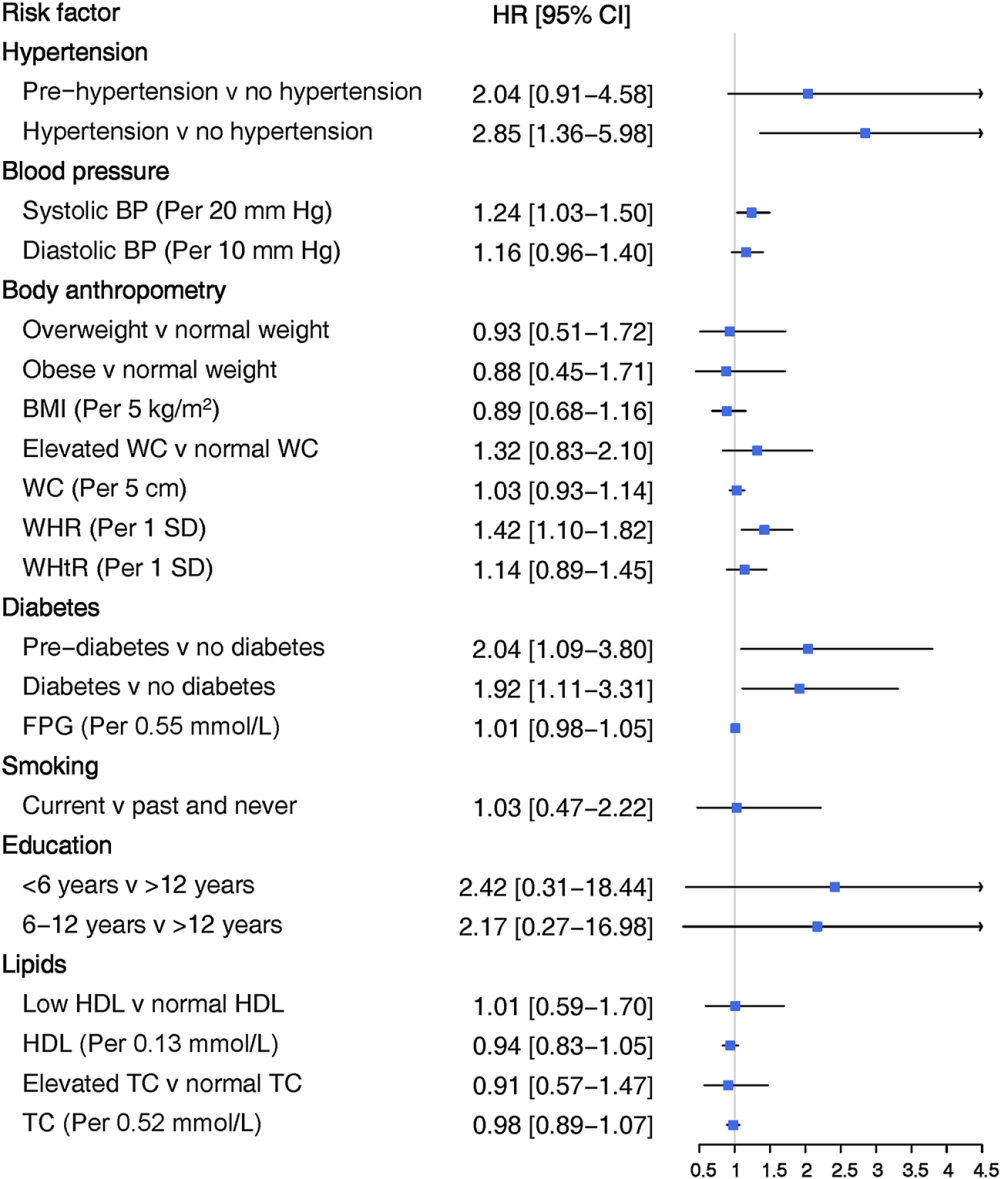
Adjusted women-to-men ratios of hazard ratios for association between risk factors and incident hard CHD. SBP: systolic blood pressure; DBP: diastolic blood pressure; BMI: body mass index; WHR: waist-to-hip ratio; WHtR: waist-to-height ratio; WC: waist circumference; FPG: fasting plasma glucose; HDL: high density lipoprotein cholesterol; TC: total cholesterol. Blood pressure-related variables were adjusted for age, smoking status, BMI, diabetes, education, TC, antihypertensive and lipid-lowering drugs, and FH-CVD. Measures of body anthropometry were adjusted for age, smoking status, SBP, diabetes, education, TC, antihypertensive and lipid-lowering drugs, and FH-CVD. Diabetes-related variables were adjusted for age, smoking status, BMI, SBP, education, TC, antihypertensive and lipid-lowering drugs, and FH-CVD. Smoking was adjusted for age, BMI, diabetes, SBP, education, TC, antihypertensive and lipid-lowering drugs, and FH-CVD. Education was adjusted for age, smoking status, BMI, diabetes, SBP, TC, antihypertensive and lipid-lowering drugs, and FH-CVD. Lipid-related variables were adjusted for age, smoking status, BMI, diabetes, SBP, education, antihypertensive and lipid-lowering drugs, and FH-CVD.

The relation between each risk factor and CHD events and associated women-to-men ratios of HRs have been shown in Figs. 1, 2, 3, and 4.

### Blood pressure

Elevated values of SBP and DBP, along with hypertension, were associated with increased risk of CHD and hard CHD in both women and men, even after adjusting for confounding variables (Figs. 1 and 3). However, hypertension was more strongly associated with the risk of hard CHD in women than men. The HR for women was 4.32 (2.16–8.65), while for men, it was 1.51 (1.07–2.14) (Fig. 3). The confounder adjusted women-to-men ratios of HRs was 2.85 (1.36–5.98) (Fig. 4).

### Anthropometric indices

A 1-SD increase in WHR was found to be associated with an increased risk of CHD in both women and men with HRs of 1.25 (1.12–1.38) and 1.15 (1.04–1.27), respectively (Fig. 1). However, this increased risk was observed specifically for hard CHD in women, with a HR of 1.62 (1.31–2.00) (Fig. 3). A 5 cm increase in WC was associated with a 5% increased risk of CHD in women, with a HR of 1.05 (1.01–1.10) (Fig. 1). Additionally, a 1-SD increment in WHtR conferred a 23% increased risk for hard CHD in women, with a HR of 1.23 (1.01–1.50) (Fig. 3). There was evidence of sex difference in the associations between WHR and hard CHD. The HRs were 1.62 (1.31–2.00) for women and 1.14 (0.97–1.34) for men (Fig. 3), resulting in a women-to-men ratio of 1.42 (1.10–1.82) (Fig. 4).

### Diabetes

Diabetes was associated with an increased risk of CHD and hard CHD in both sexes (Figs. 1 and 3). However, women with diabetes exhibited a greater risk for both CHD and hard CHD than men. The women-to-men ratio of HRs were 1.49 (1.10–2.01) (Fig. 2) and 1.92 (1.11–3.31) (Fig. 4) for CHD and hard CHD, respectively. On the other hand, pre-diabetes was only associated with an increased risk of CHD in women and conferred a greater excess risk for hard CHD in women. The women-to-men ratio of HRs for hard CHD was 2.04 (1.09–3.80) (Fig. 3). Additionally, each 0.55 mmol/L increase in FPG was associated with an increased risk of both CHD and hard CHD in both sexes (Figs. 1 and 3). However, no evidence of a sex difference was found in the association between FPG and CHD events.

### Smoking status

Compared with never/former smoking, current smoking was associated with an increased risk of both CHD and hard CHD in both sexes. The HRs were 1.56 (1.02–2.38) in women and 1.67 (1.41–1.98) in men for CHD (Fig. 1), with the corresponding values of 2.38 (1.16–4.92) and 2.31 (1.77–3.04) for hard CHD (Fig. 3). There was no evidence of a sex difference in the associations between smoking status and CHD events in confounder-adjusted models.

### Education

Compared with higher educational level (> 12 years of education), men with lower education level (6–12 years) had a 30% increased risk of CHD 1.30 (1.01–1.67) (Fig. 1). We did not find sex differences in the relation between educational level and CHD outcomes in multiple adjusted models.

### Lipids

Elevated TC was associated with more than 50% higher risk of both CHD and hard CHD in both sexes. Additionally, each 0.52 mmol/L increase in TC was associated with increased risks of approximately 10% for both CHD and hard CHD in both sexes (Figs. 1 and 3). However, an inverse association between HDL-C level and increased risk of CHD was found in both sexes. The HRs for CHD associated with low HDL-C were 1.39 (1.11–1.76) in women and 1.24 (1.05–1.47) in men (Fig. 1). The study did not find any evidence of a difference between sexes in the relationship between different lipid profiles and CHD events, after adjusting for confounders.

### Age-adjusted models

The results from the age-adjusted models for CHD and hard CHD are presented in Supplementary Tables 1 and 1, respectively. In general, the sex-specific HRs for all risk factors were slightly higher than those adjusted for multiple confounders in both CHD and hard CHD. However, in age-adjusted models, there was evidence of sex difference in the association between hypertension, SBP, FPG, and education with CHD (Supplementary Table 1). Moreover, there was an additional sex difference in the relationship between pre-hypertension and DBP with hard CHD (Supplementary Table 1).

## Discussion

In this research involving 7518 women and men aged ≥ 30 years with a median of 17.9 years follow-up, we found a crude incidence rate of 9.5 and 2.9 per 1000 person-years for CHD and hard CHD, respectively. The confounder-adjusted risk of CHD in women was less than half of that in men. Furthermore, we found that diabetes was associated with approximately 50% and 90% higher excess risk of CHD and hard CHD, respectively, in women than men. Additionally, hypertension, pre-diabetes, and increased WHR exhibited a stronger association with the risk of hard CHD in women than men. However, there was no evidence of sex differences in the relationship between smoking, lipids, education level, and CHD events.

We observed a lower crude incidence rate of CHD compared to the reported rate in Turkey (9.5 vs. 17.2 per 1000 person-years)^19^, our neighbor country in the Middle East region. However, our observed rate was higher than that reported in East Asia countries. For example, a study from Beijing, China found an age-standardized incidence of 1.6 per 1000 person-years for CHD in the overall population^20^. Similarly, another study reported rates of 1.0 and 1.8 per 1000 person-years for Japanese men and women aged 40–69 years, respectively^21^. In a different region of Iran, specifically Isfahan province, the age-standardized incidence rate of CHD were 11.7 and 8.9 per 1000 person-years for men and women, respectively^22^.

The findings from the present study add to a growing body of literature about the sex differences in risk factors for CVD. The stronger effect of hypertension on the risk of hard CHD among women is consistent with those of other large studies. The UK Biobank study found that women with hypertension had a 47% higher risk of MI compared with men with hypertension.

Using a case–control design, the INTERHEART study found higher odds ratios (OR) of MI in hypertensive women 2.95 (2.66–3.28) than in hypertensive men 2.32 (2.16–2.48)^23^. We found a 2.8 times higher relative risk of hard CHD in hypertensive women than hypertensive men which may be explained by women’s longer exposure to the effects of BP elevation. Several studies in industrialized countries have shown that BP increases faster and earlier in women than men; hence, the biological changes may impact a woman's risk for certain types of CVD begins earlier in women than men, and before they are likely thinking about their risk for CVD, resulting in poorer control and treatment of BP among women than men^24^. In a sex-specific analysis, we have previously shown that insulin resistance, as an important risk factor of CVD, had a significant role in the development of hypertension among Iranian women but not in men^25^.

In recent years, much attention has been paid to the role of gender in the association between diabetes and the risk of CVD. In the INTERHEART study, which involved 15,152 cases and 14,820 controls from 52 countries, diabetes was associated with a 4.3-fold risk for CVD events in women versus 2.7-fold in men^26^. Additionally, in a meta-analysis of 64 cohorts comprising over 800,000 individuals, a 44% higher risk of incident CHD was reported among women with diabetes compared with men with diabetes^6^. The UK Biobank study including more than 470,000 participants, found 47% greater risk for MI in women with self-reported type 2 diabetes compared with their male counterparts^27^. We extended the previous study by showing that pre-diabetes was associated with a significantly higher risk of hard CHD in women compared with men.

The stronger effect of diabetes on CHD risk in women compared with men in observational studies remains unclear. However, there is growing evidence to support the hypothesis that women experience more adverse changes in major CVD risk factors before the onset of diabetes than men^6,28^. For instance, several studies have indicated that women develop diabetes at a higher level of BMI than men^29,30^. Also, our recent study among Iranian adults aged > 20 years showed that during the transition to diabetes, women had greater adverse changes in major metabolic risk factors such as BMI, FPG, triglyceride, and HDL-C than men^31^. Similar results were also found in the Bogalusa Heart Study^28^. Therefore, the greater deterioration in CVD risk factor levels and a chronically elevated CVD risk profile in the pre-diabetic state among women may play a crucial role in sex differences observed in the association between diabetes and CHD events. Furthermore, sex differences in risk for CHD associated with diabetes have been attributed to the diversity of biological factors^32^, and sex disparity in diagnosis, management, and treatment of CVD risk factors in diabetic patients, to the detriment of women^32,33^. Recently, a Mendelian randomization study using UK Biobank data has shown that the causal effect of diabetes on the risk of CHD was similar between women and men^34^.

General and central adiposity are well recognized as the major risk factors for CHD^35^. However, there have been conflicting findings regarding the association between various anthropometric measures and the risk of CHD in both women and men^36–38^. The INTERHEART study revealed that among the different anthropometric measures, WHR exhibited the strongest correlation with the risk of MI^39^. The Nurses' Health Study reported that WHR and WC were independently associated with the risk of CHD in women^36^. Furthermore, a meta-analysis of 12 case–control studies demonstrated that a high WHR increased the risk of MI, with a greater impact observed in women compared to men^40^. Another study conducted on over 500,000 participants of the UK Biobank aged 40–69 years, found that a 1-SD higher WC and WHR conferred a higher excess risk of MI in women than in men^41^. Consistent with the findings of the INTERHEART and UK Biobank study, our study also found that WHR was more strongly associated with the risk of hard CHD in women than men.

A high WHR, as a marker of central obesity, is believed to contribute to CHD through different pathways including inflammation, oxidative stress, free fatty acids, steroid hormones, and altered production and function of adipose-derived hormone (adipokine)^40^. The greater effect of WHR on CHD among women than men may be related to the sex difference in body composition and fat distribution, with a predominance of fat mass and subcutaneous adipose tissue in women, and of muscle and visceral adipose tissue in men^41^. There is good evidence that body fat distribution is influenced by networks of sexually dimorphic genes, which are likely regulated by sex hormones^41,42^. Additionally, genome-wide association studies (GWAS) have shown that the genetic loci associated with WHR, are demonstrated to have a stronger role in women than men, containing several genes which have important roles for type 2 diabetes, lipids, and hormone metabolism^42^. Therefore, current evidence suggests some biological basis for women’s higher risk of CHD associated with WHR.

The strengths of this study are its long follow-up with a large sample size. Moreover, we examined sex differences in CHD events across a wide range of CVD risk factors in a general population from the MENA region, which has a high burden of CVD^43^. We also used the standardized methods recommended for analyzing and reporting sex differences in cardiovascular associations^44^. However, our study has some limitations. First, although we adjusted our analysis for major and well-known confounders, residual confounding due to unmeasured factors might still be possible. Secondly, our results were obtained from the population of the Tehran metropolitan area, and therefore, the findings may not be generalizable to other rural areas of Iran.

## Conclusion

This study with 20 years of follow-up showed that the risk of CHD events in women was less than half that in men. Although, several CVD risk factors, each had profound deleterious effects on the risk of CHD events in both sexes, hypertension, diabetes, pre-diabetes, and high WHR conferred a greater excess risk of CHD events in women than in men. Our findings suggest that Iranian women may require greater attention for primary or secondary prevention of CHD events.

### Supplementary Information

Supplementary Information.

## Data Availability

The datasets generated during and/or analyzed during the current study are available from the corresponding author upon reasonable request.

## References

[1] Naghavi M (2017). Global, regional, and national age-sex specific mortality for 264 causes of death, 1980–2016: A systematic analysis for the Global Burden of Disease Study 2016. Lancet.

[2] Bots SH, Peters SA, Woodward M (2017). Sex differences in coronary heart disease and stroke mortality: A global assessment of the effect of aging between 1980 and 2010. BMJ Glob. Health..

[3] Khamis RY, Ammari T, Mikhail GW (2016). Gender differences in coronary heart disease. Heart.

[4] Zhao M (2017). Sex differences in risk factor management of coronary heart disease across three regions. Heart.

[5] Bai M-F, Wang X (2019). Risk factors associated with coronary heart disease in women: A systematic review. Herz..

[6] Peters SA, Huxley RR, Woodward M (2014). Diabetes as risk factor for incident coronary heart disease in women compared with men: A systematic review and meta-analysis of 64 cohorts including 858,507 individuals and 28,203 coronary events. Diabetologia.

[7] Huxley RR, Woodward M (2011). Cigarette smoking as a risk factor for coronary heart disease in women compared with men: A systematic review and meta-analysis of prospective cohort studies. Lancet.

[8] Peters SA, Singhateh Y, Mackay D, Huxley RR, Woodward M (2016). Total cholesterol as a risk factor for coronary heart disease and stroke in women compared with men: A systematic review and meta-analysis. Atherosclerosis.

[9] Traina MI, Almahmeed W, Edris A, Tuzcu EM (2017). Coronary heart disease in the Middle East and North Africa: current status and future goals. Curr. Atheroscler. Rep..

[10] Hadaegh F (2020). Sex-specific prevalence of coronary heart disease among tehranian adult population across different glycemic status: Tehran lipid and glucose study. BMC Public. Health..

[11] Kabootari M (2018). Different weight histories and risk of incident coronary heart disease and stroke: Tehran lipid and glucose study. J. Am. Heart Assoc..

[12] Boloukat RR (2018). Impact of blood pressure, cholesterol and glucose in the association between adiposity measures and coronary heart disease and stroke among Iranian population. Clin. Nutr..

[13] Gheisari F, Emami M, Raeisi Shahraki H, Samipour S, Nematollahi P (2020). The role of gender in the importance of risk factors for coronary artery disease. Cardiol. Res. Pract..

[14] Parizadeh D, Rahimian N, Akbarpour S, Azizi F, Hadaegh F (2019). Sex-specific clinical outcomes of impaired glucose status: A long follow-up from the Tehran Lipid and Glucose Study. Eur. J. Prev. Cardiol..

[15] Azizi F, Zadeh-Vakili A, Takyar M (2018). Review of rationale, design, and initial findings: Tehran lipid and glucose study. Int J Endocrinol Metab..

[16] Chobanian AV (2003). National heart, lung, and blood institute joint national committee on prevention, detection, evaluation, and treatment of high blood pressure; national high blood pressure education program coordinating committee: the seventh report of the joint national committee on prevention, detection, evaluation, and treatment of high blood pressure: The JNC 7 report. JAMA.

[17] Hadaegh F (2009). Appropriate cutoff values of anthropometric variables to predict cardiovascular outcomes: 7.6 years follow-up in an Iranian population. Int. J. Obes..

[18] Khalili D (2018). Outcomes of a longitudinal population-based cohort study and pragmatic community trial: findings from 20 years of the Tehran Lipid and Glucose Study. Int. J. Endocrinol. Metab..

[19] Onat A (2010). High absolute coronary disease risk among Turks: Involvement of risk factors additional to conventional ones. Cardiology.

[20] Sun J (2012). Surveillance on the incidence of acute coronary events in the permanent residents of Beijing aged 25 years and more from 2007 to 2009. Zhonghua Xin Xue Guan Bing Za Zhi.

[21] Kitamura A (2008). Trends in the incidence of coronary heart disease and stroke and their risk factors in Japan, 1964 to 2003: The Akita-Osaka study. J. Am. Coll. Cardiol..

[22] Talaei M (2013). Incidence of cardiovascular diseases in an Iranian population: The Isfahan Cohort Study. Arch. Iran. Med..

[23] Anand SS (2008). Risk factors for myocardial infarction in women and men: Insights from the INTERHEART study. Eur. Heart J..

[24] Ji H (2020). Sex differences in blood pressure trajectories over the life course. JAMA Cardiol..

[25] Arshi B (2015). Sex-specific relations between fasting insulin, insulin resistance and incident hypertension: 8.9 years follow-up in a Middle-Eastern population. J. Hum. Hypertens..

[26] Yusuf S (2004). Effect of potentially modifiable risk factors associated with myocardial infarction in 52 countries (the INTERHEART study): Case-control study. Lancet.

[27] Millett ER, Peters SA, Woodward M (2018). Sex differences in risk factors for myocardial infarction: cohort study of UK Biobank participants. Brit. Med. J..

[28] Du T (2019). Sex differences in cardiovascular risk profile from childhood to midlife between individuals who did and did not develop diabetes at follow-up: the Bogalusa Heart Study. Diabetes Care.

[29] Paul S, Thomas G, Majeed A, Khunti K, Klein K (2012). Women develop type 2 diabetes at a higher body mass index than men. Diabetologia.

[30] Wannamethee S (2012). Do women exhibit greater differences in established and novel risk factors between diabetes and non-diabetes than men? The British Regional Heart Study and British Women’s Heart Health Study. Diabetologia.

[31] Ramezankhani A, Azizi F, Hadaegh F (2020). Sex differences in rates of change and burden of metabolic risk factors among adults who did and did not go on to develop diabetes: two decades of follow-up from the Tehran lipid and glucose study. Diabetes Care.

[32] Peters SA, Huxley RR, Sattar N, Woodward M (2015). Sex differences in the excess risk of cardiovascular diseases associated with type 2 diabetes: Potential explanations and clinical implications. Curr. Cardiovasc. Risk Rep..

[33] Xu G (2019). Risk of all-cause and CHD mortality in women versus men with type 2 diabetes: A systematic review and meta-analysis. Eur. J. Endocrinol..

[34] Peters TM (2021). Sex differences in the risk of coronary heart disease associated with type 2 diabetes: A Mendelian randomization analysis. Diabetes Care.

[35] Dale CE (2017). Causal associations of adiposity and body fat distribution with coronary heart disease, stroke subtypes, and type 2 diabetes mellitus: A Mendelian randomization analysis. Circulation.

[36] Rexrode KM (1998). Abdominal adiposity and coronary heart disease in women. JAMA.

[37] Page JH (2009). Waist-to-height ratio as a predictor of coronary heart disease among women. Epidemiology.

[38] Nguyen NT, Nguyen X-MT, Wooldridge JB, Slone JA, Lane JS (2010). Association of obesity with risk of coronary heart disease: findings from the National Health and Nutrition Examination Survey, 1999–2006. Surg. Obes. Relat. Dis..

[39] Yusuf S (2005). Obesity and the risk of myocardial infarction in 27 000 participants from 52 countries: A case-control study. Lancet.

[40] Cao Q (2018). Waist-hip ratio as a predictor of myocardial infarction risk: A systematic review and meta-analysis. Medicine.

[41] Peters SA, Bots SH, Woodward M (2018). Sex differences in the association between measures of general and central adiposity and the risk of myocardial infarction: results from the UK Biobank. J. Am. Heart Assoc..

[42] Randall JC (2013). Sex-stratified genome-wide association studies including 270,000 individuals show sexual dimorphism in genetic loci for anthropometric traits. PLoS Genet..

[43] Azizi F (2019). Metabolic health in the middle east and north Africa. Lancet Diabetes Endocrinol..

[44] Woodward M (2019). Rationale and tutorial for analysing and reporting sex differences in cardiovascular associations. Heart.

