# Supplementary material for: Sex differences in risk factors for coronary heart disease events: a prospective cohort study in Iran
Source: Sci Rep. 2023 Dec 16;13:22398. doi: 10.1038/s41598-023-50028-0 (PMC10725458; doi:10.1038/s41598-023-50028-0)
Supplement: Supplementary file 1 — Supplementary Information. [file 41598_2023_50028_MOESM1_ESM.pdf]

Sex differences in risk factors for coronary heart disease events: a prospective cohort study in Iran

Azra Ramezankhani[1], Fereidoun Azizi[2], Farzad Hadaegh[1,*]

[1]Prevention of Metabolic Disorders Research Center, Research Institute for Endocrine Sciences, Shahid Beheshti University of Medical Sciences, Tehran, Iran

[2]Endocrine Research Center, Research Institute for Endocrine Sciences, Shahid Beheshti University of Medical Sciences, Tehran, Iran

***Correspondence to:**
Farzad Hadaegh, MD, Professor of Internal Medicine and Endocrinology
Address: Prevention of Metabolic Disorders Research Center, Research Institute for Endocrine Sciences, Shahid Beheshti University of Medical Sciences
Number 24, Yemen Street, Shahid Chamran Highway
P.O. Box: 19395-4763, Tehran, Iran
E-mail: fzhadaegh@endocrine.ac.ir
Phone: +982122432500
Fax: +982122416264

SUPPLEMENTAL MATERIAL

Supplementary Table 1 Baseline characteristics of respondents and non-respondents

Characteristics	Non-respondents n=1433	Respondents n=7518	P value
**Men, n (%)**	634 (44.2)	3377 (44.9)	0.643
**Age,** year	46.97 (13.46)	46.68 (12.08)	0.455
**Blood pressure, mm Hg**			
SBP	121.21 (19.81)	121.19 (19.64)	0.970
DBP	78.52 (10.71)	78.59 (10.99)	0.718
**Hypertension, n (%)**			
No hypertension	500 (40.7)	3104 (41.3)	
Pre-hypertension	427 (34.8)	2549 (33.9)	0.828
Hypertension	300 (24.4)	1865 (24.8)	
**BMI categories, n (%)**			
Normal weight	373 (32.4)	2252 (30.0)	
Overweight	499 (43.4)	3249 (43.2)	0.105
Obese	279 (24.2)	2017 (26.8)	
**Body anthropometry**			
BMI, kg/m^2	27.19 (4.67)	27.52 (4.56)	0.024
WC, cm	89.61 (11.43)	90.82 (11.53)	0.001
Waist-to-hip ratio	0.88 (0.08)	0.89 (0.08)	0.024
Waist-to-height ratio	0.55 (0.07)	0.56 (0.07)	0.004
**WC categories, n (%)**			
Normal WC	747 (66.0)	4706 (62.6)	0.013
Elevated WC	384 (34.0)	2812 (37.4)	
**Diabetes status, n (%)**			
No diabetes	603 (61.2)	4786 (63.7)	
Pre-diabetes	226 (22.9)	1733 (23.1)	0.070
Diabetes	157 (15.9)	999 (13.3)	
**FPG, (mmol/l)**	5.58 (2.17)	5.59 (1.93)	0.980
**Smoking status, n (%)**			
Past and never	976 (78.7)	6252 (83.2)	<0.001
Current	264 (21.3)	1266 (16.8)	
**Education level, n (%)**			
<6 years	563 (39.7)	2917 (38.8)	
6-12 years	682 (48.1)	3674 (48.9)	0.813
>12 years	173 (12.2)	927 (12.3)	
**Lipids categories, n (%)**			
Normal HDL-C	335 (29.0)	2219 (29.5)	0.382
Low HDL-C	819 (71.0)	5299 (70.5)	
Normal TC	845 (72.5)	5616 (74.7)	0.062
Elevated TC	320 (27.5)	1902 (25.3)	
**Lipids measures, (mmol/l)**			
HDL-C	1.09 (0.29)	1.07 (0.28)	0.009
TC	5.55 (1.19)	5.52 (1.17)	0.381
**Drug use, n (%)**			
Antihypertensive drugs	108 (7.5)	583 (7.8)	0.413
Lipid lowering drugs	63 (4.4)	275 (3.7)	0.104
**Family history of CVD, n (%)**	211 (14.7)	1227 (16.3)	0.070

SBP: systolic blood pressure; **DBP**: diastolic blood pressure; **BMI**: body mass index; **WHR**: waist-to-hip ratio; **WHtR**: waist-to-height ratio; **WC**: waist circumference; **FPG**: fasting plasma glucose; **HDL-C**: high density lipoprotein cholesterol; **TC**: total cholesterol; **CVD:** cardiovascular diseases

Supplementary Table 2 Age adjusted HRs for incident CHD associated with risk factors, by sex

	Women HR (95% CI)	Men HR (95% CI)	women-to-men HRs (95% CI)
**Hypertension**			
Pre-hypertension *v* no hypertension	1.46 (1.10-1.94)	1.17 (0.96-1.42)	1.25 (0.88-1.76)
Hypertension *v* no hypertension	2.78 (2.14-3.61)	1.71 (1.40-2.10)	1.62 (1.18-2.23)
**Blood pressure**			
SBP (Per 20 mmHg)	1.41 (1.31-1.53)	1.27 (1.18-1.37)	1.11 (1.01-1.23)
DBP (Per 10 mmHg)	1.28 (1.18-1.39)	1.19 (1.11-1.27)	1.07 (0.97-1.19)
**Body mass index**			
Overweight *v* normal weight	1.52 (1.14-2.02)	1.43 (1.20-1.71)	1.06 (0.76-1.47)
Obese *v* normal weight	1.68 (1.27-2.23)	1.45 (1.15-1.83)	1.15 (0.80-1.66)
BMI (Per 5 kg/m^2)	1.18 (1.07-1.30)	1.24 (1.13-1.37)	0.94 (0.82-1.08)
**Waist circumference**			
Elevated WC *v* normal WC	1.44 (1.20-1.75)	1.37 (1.17-1.61)	1.05 (0.82-1.34)
WC (Per 5 cm)	1.14 (1.09-1.18)	1.10 (1.06-1.14)	1.03 (0.98- 1.09)
**Waist-to-hip ratio**			
WHR (Per 1 SD)	1.49 (1.35-1.65)	1.33 (1.22-1.46)	1.12 (0.98-1.27)
**Waist-to-height ratio**			
WHtR (Per 1 SD)	1.38 (1.26-1.52)	1.27 (1.16-1.39)	1.08 (0.95-1.23)
**Diabetes**			
Pre-diabetes *v* no diabetes	1.63 (1.28-2.08)	1.20 (1.00-1.45)	1.35 (0.99-1.83)
Diabetes *v* no diabetes	3.91 (3.13-4.88)	2.08 (1.70-2.55)	1.87 (1.39-2.52)
**Fasting plasma glucose**			
FPG (Per 0.55 mmol/L)	1.09 (1.08-1.11)	1.07 (1.05-1.08)	1.02 (1.00-1.04)
**Smoking**			
Current *v* past and never	1.42 (0.94-2.17)	1.56 (1.31-1.84)	0.91 (0.58-1.43)
**Education**			
<6 years v >12 years	2.83 (1.45-5.53)	1.03 (0.79-1.35)	2.75 (1.35-5.59)
6-12 years *v* >12 years	1.84 (0.93-3.64)	1.38 (1.07-1.76)	1.33 (0.64-2.76)
**Lipids**			
Low HDL-C *v* normal HDL-C	1.46 (1.16-1.84)	1.39 (1.17-1.64)	1.05 (0.79-1.39)
HDL-C (Per 0.13 mmol/L)	0.91 (0.87-0.95)	0.91 (0.87-0.95)	1.00 (0.93-1.06)
Elevated TC *v* normal TC	1.80 (1.49-2.18)	1.84 (1.56-2.18)	0.98 (0.75-1.26)
TC (Per 0.52 mmol/L)	1.15 (1.12-1.19)	1.15 (1.11-1.19)	1.00 (0.95-1.05)

BP: blood pressure; **SBP**: systolic BP; **BMI**: body mass index; **WHR**: waist-to-hip ratio; **WHtR**: waist-to-height ratio; **WC**: waist circumference; **FBS**: fasting plasma glucose; **HDL**: high density lipoprotein; **TC**: total cholesterol; **CVD**: cardiovascular diseases; **FH-CVD:** family history of CVD; **HR**: hazard ratio; **CI**: confidence interval
Mean (SD) of WHR: 0.89 (0.08) ; Mean (SD) of WHtR: 0.56 (0.07)

Supplementary Table 3 Age adjusted HRs for incident hard CHD associated with risk factors, by sex

	Women HR (95% CI)	Men HR (95% CI)	women-to-men HRs (95% CI)
**Hypertension**			
Pre-hypertension *v* no hypertension	2.20 (1.05;4.61)	0.89 (0.64-1.24)	2.46 (1.10-5.51)
Hypertension *v* no hypertension	5.92 (3.02-11.59)	1.43 (1.04-1.98)	4.13 (1.99-8.56)
**Blood pressure**			
SBP (Per 20 mmHg)	1.65 (1.43-1.91)	1.18 (1.04-1.33)	1.40 (1.17-1.67)
DBP (Per 10 mmHg)	1.47 (1.26-1.72)	1.14 (1.02-1.27)	1.29 (1.07-1.56)
**Body mass index**			
Overweight *v* normal weight	1.22 (0.71-2.08)	1.30 (0.97-1.73)	0.93 (0.51-1.72)
Obese *v* normal weight	1.22 (0.71-2.10)	1.48 (1.02-2.14)	0.82 (0.42-1.58)
BMI (Per 5 kg/m^2)	1.09 (0.89-1.33)	1.27 (1.09-1.48)	0.85 (0.66-1.10)
**Waist circumference**			
Elevated WC *v* normal WC	1.82 (1.23-2.68)	1.33 (1.03-1.71)	1.36 (0.86-2.17)
WC (Per 5 cm)	1.16 (1.07-1.26)	1.11 (1.04-1.17)	1.05 (0.95-1.16)
**Waist-to-hip ratio**			
WHR (Per 1 SD)	1.94 (1.59-2.37)	1.31 (1.13-1.53)	1.47 (1.15-1.88)
**Waist-to-height ratio**			
WHtR (Per 1 SD)	1.50 (1.24-1.81)	1.27 (1.09-1.48)	1.17 (0.92-1.49)
**Diabetes**			
Pre-diabetes *v* no diabetes	1.71 (1.01-2.89)	0.73 (0.52-1.02)	2.35 (1.26-4.38)
Diabetes *v* no diabetes	4.70 (2.98-7.39)	1.92 (1.41-2.62)	2.44 (1.42-4.19)
**Fasting plasma glucose**			
FPG (Per 0.55 mmol/L)	1.10 (1.08-1.13)	1.07 (1.05-1.10)	1.02 (0.99-1.06)
**Smoking**			
Current *v* past and never	2.13 (1.04-4.39)	2.12 (1.62-2.76)	1.01 (0.46-2.16)
**Education**			
<6 years v >12 years	5.55 (0.77-40.08)	1.47 (0.91-2.37)	3.76 (0.49-28.51)
6-12 years *v* >12 years	3.71 (0.50-27.40)	1.60 (1.01-2.53)	2.32 (0.29-18.09)
**Lipids**			
Low HDL *v* normal HDL	1.27 (0.81-1.98)	1.36 (1.03-1.79)	0.92 (0.55-1.57)
HDL-C (Per 0.13 mmol/L)	0.90 (0.82-0.98)	0.92 (0.86-0.99)	0.97 (0.86-1.09)
Elevated TC *v* normal TC	1.81 (1.23-2.66)	1.76 (1.34-2.31)	1.02 (0.64-1.64)
TC (Per 0.52 mmol/L)	1.15 (1.07-1.22)	1.13 (1.07-1.19)	1.01 (0.93-1.10)

BP: blood pressure; **SBP**: systolic BP; **BMI**: body mass index; **WHR**: waist-to-hip ratio; **WHtR**: waist-to-height ratio; **WC**: waist circumference; **FBS**: fasting plasma glucose; **HDL**: high density lipoprotein; **TC**: total cholesterol; **CVD**: cardiovascular diseases; **FH-CVD:** family history of CVD; **HR**: hazard ratio; **CI**: confidence interval
Mean (SD) of WHR: 0.89 (0.08) ; Mean (SD) of WHtR: 0.56 (0.07)

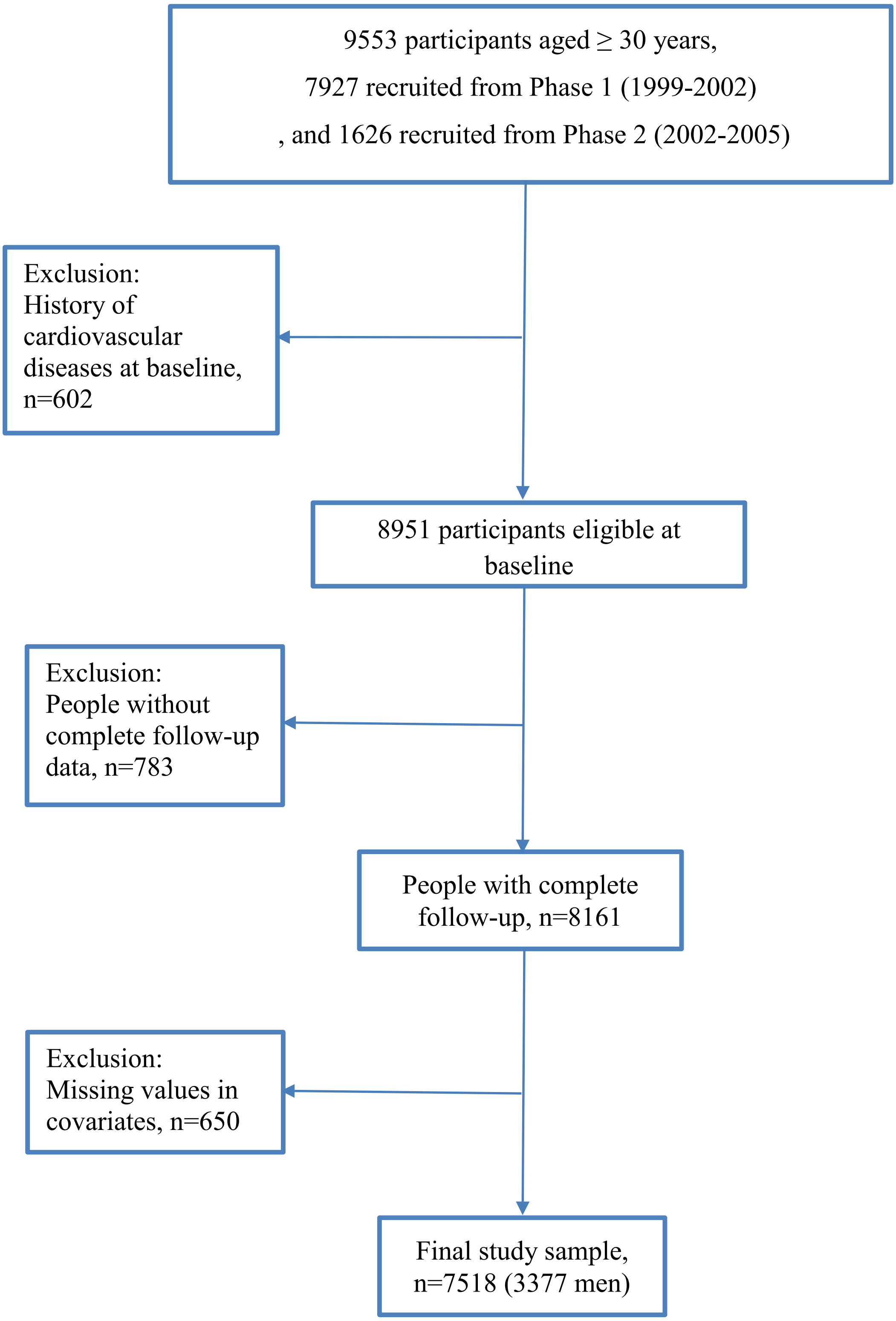

Supplementary Figure 1: Flowchart of sample selection for the study
